# Psychological well-being and L1 learning grit among Ghanaian language students in higher education: a PLS-SEM analysis

**DOI:** 10.3389/fpsyg.2026.1791930

**Published:** 2026-03-18

**Authors:** Ernest Nyamekye, Abdul-Rahman Mutawakil

**Affiliations:** Department of Arts Education, University of Cape Coast, Cape Coast, Ghana

**Keywords:** Ghanaian language, higher education, L1 grit, language learning, PLS-SEM, well-being

## Abstract

**Introduction:**

Scholars in the field of L1 education in Ghana have argued that students pursuing Ghanaian languages as a program of study at the higher education level pass through psychological burdens to achieve their learning goals. It has been argued that these psychological burdens, which probably stem from the negative attitudes among peers and lecturers, coupled with limited institutional support for students pursuing Ghanaian languages, may have a detrimental effect on the effort they put into their learning. Since these scholarly suppositions lack enough empirical backing, the current study aimed to explore the psychological wellbeing of students and its influence on the L1 learning grit (LLG) using a partial least squares structural equation modeling approach.

**Methods:**

A census survey was used to recruit 173 Ghanaian language students in two higher educational institutions in the Cape Coast metropolis of the Central region of Ghana. Partial least squares structural equation modelling was used to analysis the data.

**Results:**

The study revealed that psychological well-being variables including self-efficacy, and positive learning experience are significant predictors of students’ L1 grit. Overall satisfaction and enjoyment of L1 education, emotional stability and management, and social connectedness were not significant predictors of students’ L1 grit.

**Conclusion:**

It can be argued that, in the context of indigenous language learning in Ghana, the strongest determinant of students’ learning grit is their sense of confidence and positive learning experience. However, emotional stability, overall satisfaction, and social connectedness do not influence grit, and this is attributable to the constant marginalization of indigenous languages in educational domains.

## 1 Introduction

Language remains very significant to education, economic development and the cultural identity of people from all walks of life (Nyamekye et al., 2023; Sabirova and Tenkorang, 2021). Proficiency in one’s native language (L1) is connected to academic success, professional growth, and national cohesion (Cummins, 1978; Parmegiani, 2019, 2022). Yet, the challenges with language learning in Ghana remains enormous (Nyamekye, 2024). Many students, language learners precisely, enter university with great zeal and determination to study a language, perhaps driven by the prestige, in the case of foreign languages and/or cultural connection and personal growth in the case of local languages. This enthusiasm is mostly refurbished by promising career opportunities that language proficiency can offer. However, after some time they may struggle to be committed, feel frustrated and eventually withdraw—what scholars call attrition (Damron and Forsyth, 2012; Wesely, 2010). Psychological and emotional burdens appear to be among the major root causes of this frustration and eventual withdrawal among students (Nguyen and Tran, 2023; Wang and Zhang, 2021). Consequently, the same psychological and emotional burden (incited by disrepute and infamy) associated with indigenous language learning are regarded as obstacles to language learning in Ghana (Asamoah, 2022; Nyamekye, 2024; Owu-Ewie and Edu-Buandoh, 2014). Aside from the immersive language environment the Ghanaian universities come with, academic pressure, fear of making mistakes, and societal attitudes—particularly toward indigenous languages— stand tall as challenges to language learning (Asamoah, 2022; Dansieh et al., 2021). These challenges, among others, leave learners of these languages lowly motivated, highly anxious, and eventually, disengaging. In the cases of indigenous languages, it appears worse, as those languages are regarded economically invaluable as compared to English and other foreign languages (Amponsah and Koga, 2022; Nyamekye, 2024; Owu-Ewie and Edu-Buandoh, 2014). This leads to psychological distress, which would make some learners of these languages abandon their language-learning efforts, albeit others exhibit remarkable perseverance (grit) regardless.

The nexus between psychological well-being and the perseverance and vehement passion for ambitions and/or long-term goals which is termed grit seems underexplored in the context of languages as far as Ghana is concerned, albeit it is widely studied in education. As studies suggest, psychological well-being, which has embedded in it, self-efficacy, mental resilience, and emotional stability, has a significant impact on one’s ability to persist regardless (Bada et al., 2020; Sisto et al., 2019). This is to say that while poor psychological well-being may lead to frustration, avoidance and eventual withdrawal, the opposite remains true, i.e., good or positive psychological well-being may enhance zeal, reduce anxiety, and promote resilience. As aforesaid, the paucity of literature in the Ghanaian context as far as the interaction between grit and psychological factors within the pretext of indigenous language learning makes this study timely and relevant. This study therefore aims to explore the predictive impact of psychological wellbeing on the language learning grit among students pursuing indigenous languages in higher education.

## 2 Literature review

### 2.1 Conceptual review

#### 2.1.1 Operationalization of L1 learning grit

Grit is a concept traced to Duckworth et al. (2007). Conceptualized as a personal trait, grit simply has to do with one’s ability to sustain both effort and interest in pursuits that require substantial time, resilience and/or enviable commitment (Duckworth and Quinn, 2009). Grit, as suggested by empirical evidence, is positively correlated with numerous indicators of success, including educational attainment, job retention and even military training completion rates (Eskreis-Winkler et al., 2014; Robertson-Kraft and Duckworth, 2014; Suzuki et al., 2015). Long-term stamina, perseverance, and unwavering commitment to a singular goal are what grit emphasizes. Grit, according to Duckworth and Quinn (2009), comprises two dimensions, namely, perseverance of effort and consistency of interest. An individual’s capacity to persist through hurdles or challenges is what is refer to as perseverance of effort, while one’s capacity to stay very committed to long-term goals without frequent changes in aspirations is termed consistency of interest.

Consequently, grit has been associated with increased student engagement, higher academic achievement, and greater aspirations for long-term achievement within the educational context (Clark and Malecki, 2019; Duckworth and Quinn, 2009; Strayhorn, 2014). In language learning, long-term commitment, consistence practice, and resilience are much required to attain success. Somewhat different from other academic subjects, the cumulative nature of language learning (Berkes et al., 2012) coupled with repeated failures, cognitive overloads and psychological/ affective setbacks such as anxiety (Chen and Chang, 2009; Wang et al., 2024), makes grit a crucial factor in achieving success thereof. Moreover, within the Ghanaian landscape, indigenous language learning presents unique challenge including sociolinguistic attitudes, institutional supports, and psychological setbacks, making the psychological well-being of students crucial in sustaining their motivation and interest (Akele Twumasi, 2021; Nyamekye, 2024). The dominance of English as the medium of instruction coupled with the stigma surrounding indigenous language learning in formal education has resulted in a drastic reduction in enthusiasm for indigenous language learning (Djorbua et al., 2024; Nyamekye, 2024; Owu-Ewie and Edu-Buandoh, 2014). This suggests that, within the domain of indigenous language learning in Ghana the concept of grit really need exploration. This is because, the situations of stigma and what not surrounding indigenous language projects serious psychological stress and/or low self-efficacy that may impact the required perseverance required to commit to indigenous language mastery, albeit those with enviably higher resilience and motivation (grit) may persist regardless.

#### 2.1.2 Psychological well-being

Basically, psychological well-being has to do with a person’s overall mental state being sound. This encapsulates cognitive functioning, personal fulfillment, emotional stability, and resilience (Gautam et al., 2024; Levine et al., 2021). For its multidimensional nature, it travers beyond the mere absence of distress or illness, rather, it includes that one has self-actualization, purpose, autonomy and personal growth (Ryff and Singer, 1998; Winefield et al., 2012). This is to say that as far as the psychological well-being of an individual is concerned, positive and negative effects are independent dimensions, thus, rather than existing on a simple spectrum of happiness versus distress, an individual can actually experience significantly higher levels of both (Bradburn, 1969). To wit, psychological well-being is not just the absence of mental illness, stress or anxiety but, to that, the presence of self-efficacy, contentment, engagement and positive emotions.

Using the framework of Renshaw et al. (2015), students psychological well-being covers students covers these subconstructs: positive learning experience, self-efficacy, social connectedness, emotional stability and management, and overall satisfaction and enjoyment. Positive learning experience as a wellbeing subdimension concerns students’ perception of how their classroom experiences are meaningful, engaging and supportive of their academic growth, while self-efficacy concerns their sense of confidence in their ability to accomplish their academic task. Social connectedness is also considered an important part of their wellbeing because it concerns sense of belonging and quality relationships within the school environment, including their peers, teachers and administrators. Emotional management and stability on the other hand concerns student’s ability to regulate their emotional responses effectively in social context while their overall satisfaction and enjoyment captures general sense of happiness and fulfillment with their school life (Renshaw et al., 2015). Exploring these dimensions offer an opportunity to comprehensively understand the psychological states of students.

Psychological well-being is very crucial to academic success, especially language learning, within which interest, resilience, and sustained efforts cannot be overemphasized. As studies have shown, this is to say that individuals with psychological well-being, for the fact that they experience, sound emotional regulation, social connectedness and greater life satisfaction, would attain higher academic performance and personal achievement (Mustafa et al., 2020; Yu et al., 2018). Moreover, psychological well-being is linked to motivation, concentration, problem-solving abilities and reasonable levels of cognitive flexibility (Seligman, 2002; Tong et al., 2024). Conversely, students lacking psychological well-being, perhaps experiencing stress, anxiety, and negative emotions, are likely to experience low academic success as they would struggle to sustain their efforts, stay motivated, persevere, and retain their memory (Pascoe et al., 2020; Youcefi et al., 2023; Zhang et al., 2024).

### 2.2 Theoretical framework and hypotheses

In the context of the link between psychological wellbeing and language learning grit is anchored in the self-determination theory (SDT), a theory developed by Ryan and Deci (2024). The theoretical position of the SDT foregrounds the need for understanding the connection between psychological well-being and language learning grit. SDT posit that psychological wellness is a prerequisite for motivation and sustained effort among individuals (Gagné and Deci, 2005). The theory particular holds that when individuals experience emotional stability and wellbeing, they tend feel competence and self-directed, hence improving the autonomy and sustaining the effort in pursuit of a given goal even in times of difficulty. This means that individuals who are psychologically unstable are likely to undermine persistence required for a long-term goal pursuit.

Over the years, a growing body of literature have declaimed the interconnectedness of grit and psychological well-being, and most of these studies revealed that grit incites life satisfaction and subjective happiness, and as well, makes individuals emotionally stable (Choi, 2020; Datu et al., 2016; Li et al., 2018; Saunders et al., 2023; Von Culin et al., 2014). Drawing from this existing literature, we say gritty individuals are able to persist regardless in academic and professional domains, especially in language learning, due to their ability to manage stress more effectively and, thereby, experience lower levels of anxiety while cultivating an intrinsic sense of accomplishment. For instance, Lake (2013) revealed that gritty students portrayed higher levels of sound psychological well-being as they exhibited strong passion and persistence in language learning and portrayed a sense of self-esteem, happiness, and curiosity. Similarly, Wei et al. (2019) found that emotional well-being —particularly language learning enjoyment— significantly influenced the relationship between grit and academic success. Also, Changlek and Palanukulwong (2015) reported that students who exhibited lower levels of language anxiety and higher levels of perseverance attained high achievement in language learning.

All the studies above looked at psychological well-being and L2 grit. Even though empirical data has established that L1 learning in Ghana comes with a lot of emotional and psychological challenges, there seems to be a paucity of literature on the nexus between psychological well-being and L1 grit. Against this backdrop the current study seeks to explore the psychological well-being and L1 grit —in this study, referred to as LLG — of Ghanaian university students. To achieve this sole objective, the study is guided by the following hypothesis:

*H1*: Emotional stability and management will positively influence students’ LLG.

*H2*: Overall satisfaction and enjoyment will positively influence students’ LLG.

*H3*: Positive learning experience will positively influence students’ LLG.

*H4*: Social connectedness will positively influence students’ LLG.

*H5*: Self-efficacy will positively influence students’ LLG.

## 3 Materials and methods

### 3.1 Research designs and participants

This study, being a quantitative study, employed a cross-sectional survey design. Though this particular research design comes with some challenges or disadvantages, it was considered for this study for its flexibility and allowance for reaching a large number of participants within a considerably short period of time (Connelly, 2016). Most notably, this nature the chosen design come with would help attain the generalizability advantage quantitative studies come with Borgstede and Scholz (2021). The participants of the current study were recruited via a nonprobability sampling technique, specifically, the convenient sampling method. For the fact that the population of the study was all L1 students in two higher educational institutions in the Cape Coast metropolis (University of Cape Coast and OLA College of Education), convenient sampling was deemed the best technique to recruit participants to be included in the current study. This allowed us reach participants based on availability and readiness. Though it can be argued, like any other sampling technique, that convenient sampling technique come with some drawbacks, it provides a satisfactory sample (Golzar et al., 2022).

### 3.2 Instrumentation

The instrument for the current study, structured on a five-point Likert scale, had three sections; section A took demographic background of the respondents, B took data of LLG of the respondents, and C took data for PWB of the respondents. The scale used to gather data on the PWB of students were adapted from the student psychological well-being scale of Renshaw et al. (2015). This instrument had constructs that align with the current sub-scales positive learning experience, self-efficacy, social connectedness, emotional stability and management, and overall satisfaction and enjoyment. Each of these five scales had five items with questions like ‘Learning Ghanaian Language makes me feel excited and motivated’ for Learning Experience, ‘I am confident in my ability to improve my Ghanaian Language skills’ for Self-Efficacy, ‘I feel connected to my classmates and enjoy learning Ghanaian Language with them’ for Social Connectedness, ‘I don’t let mistakes in Ghanaian Language class affect my confidence’ for Emotional Stability and Management, and ‘Learning the Ghanaian Language brings a sense of purpose and meaning to my life’ for Overall Satisfaction and Enjoyment.

The instrument that was used to gather data on LLG was adapted from the Grit Scale of Duckworth et al. (2007). The scale has two sub-scales, i.e., Consistency of interest and Perseverance of effort. According to Duckworth and Quinn (2009), consistency of interest has to do with ones capacity to stay very commitment to long-term goals without frequent changes in aspirations while perseverance of effort has to do with an individual’s capacity to persist through hurdles or challenges. For each sub scale of the LLG instrument, there were five items with questions like ‘Even when Ghanaian Language grammar is challenging, I don’t give up easily’ for perseverance of effort, ‘I don’t lose interest in learning even if I face repeated difficulties’ consistency of interest, ‘When I make mistakes in Ghanaian Language, I try again instead of giving up’.

### 3.3 Data collection and analysis

Data was collected through the administration of questionnaires. The gathered data was then coded and cleaned in SPSS and later analyzed with Smart PLS 4. With the partial least squares structural equation modeling (PLS-SEM) approach, the measurement model was assessed to determine the adequacy and structural quality of the measurements. This process included an assessment of the internal consistency of the measurements as well as the convergent and discriminant validity of the measurements. In addition to that, to examine the existence of collinearity within the measure model, common method bias was also used. Using the PLS-SEM bootstrap technique, the hypotheses proposed in the current study were then tested after ascertaining the quality of the measurement model.

### 3.4 Ethical consideration

For the fact that the participants of the study were humans, strict research ethics were put in place. The study followed the ethical protocols that aligned with the Declaration of University of Cape Coast’s Institutional Review Board. All participants gave their informed consent to participate in the study before being able to fill in the questionnaire. Following this standard, an ethical clearance from the Institutional Review Board of the University of Cape Coast was obtained. Also, the consent of participants was sought. We also ensured that the data gathered were used solely for the study while ensuring no third party accessed it. Moreover, no participant was forced in any way or form to partake on this study.

## 4 Results

### 4.1 Demographic data

The background information of the participants of the current study were discussed in this section. This background information included gender, age range, type of student, institution type, among others. In Table 1, a summary of the demographic information of the participant of this study is presented.

**Table 1:** Demographic data (*N* = 173).

Variable		Frequency	Percent
Gender	Male	53	30.6
	Female	120	69.4
Age Range	10–20	19	11.0
	21–30	154	89.0
Minor or Major	Major	121	69.9
	Minor	52	30.1
Type of Student	Education students	145	83.8
	Non-education students	28	16.2
Institution	College of Education	104	60.1
	University	69	39.9

Source: Field data 2025.

From Table 1, it is identified that more than half of the study participants (120) who represents 69.4% are females. With only 53 (30.6) males, we realize that the female participants formed the greatest percentage in the current study. Perhaps females enroll more in Ghanaian language programs at the tertiary levels as compared to males. With regards to age range, we recorded the greatest percentage (89%; 154) being between the age range of 21–30 with only 11% (19) falling within the ages of 10–20. Though the instrument employed for data collection had the age range of 31–40 and Above 40, no participant recorded being within those age ranges. When this happens, SPSS automatically would not present that in the output, the reason those age ranges (31–40 and Above 40) do not appear in Table 1. Moreover, it was inquired whether the participants of the study major (specialize) in the said Ghanaian language or only take some courses in it (minor). On that, it was recorded that the majority (121) who make up 69.9 percent specialize, i.e., major Ghanaian language with the remaining 52 (30.1%), only reading some courses in Ghanaian language.

Again, it was inquired whether the participants are education students, viz., student teachers, or not. It was found with regards to this that the enviable majority (145) who represents 83.8% of the total participants were education students. The remaining 28 who also made-up 16.2% percent were not education students. Lastly, we sought to know the institution type of the participants, wherein, it was found that the overwhelming majority (104; 60.1%) were in the colleges of education. Only 69 (39.9) participants recorded to be in the University. Perhaps most university student do not peruse Ghanaian languages as compared to the colleges of Education. These findings provide us with a better picture of the nature and/or characteristics of the participants of the current study.

### 4.2 Measurement model evaluation

In PLS-SEM analysis, researchers are required to evaluate the adequacy of the measurements to lend credibility to the results of the hypotheses. Evaluating the measurement model involves running reliability, convergent validity, discriminant validity, and common method bias assessment. The following subsections presents results of the measurement quality based on the aforementioned statistical criterion.

#### 4.2.1 Reliability and convergent validity

**Figure 1:**
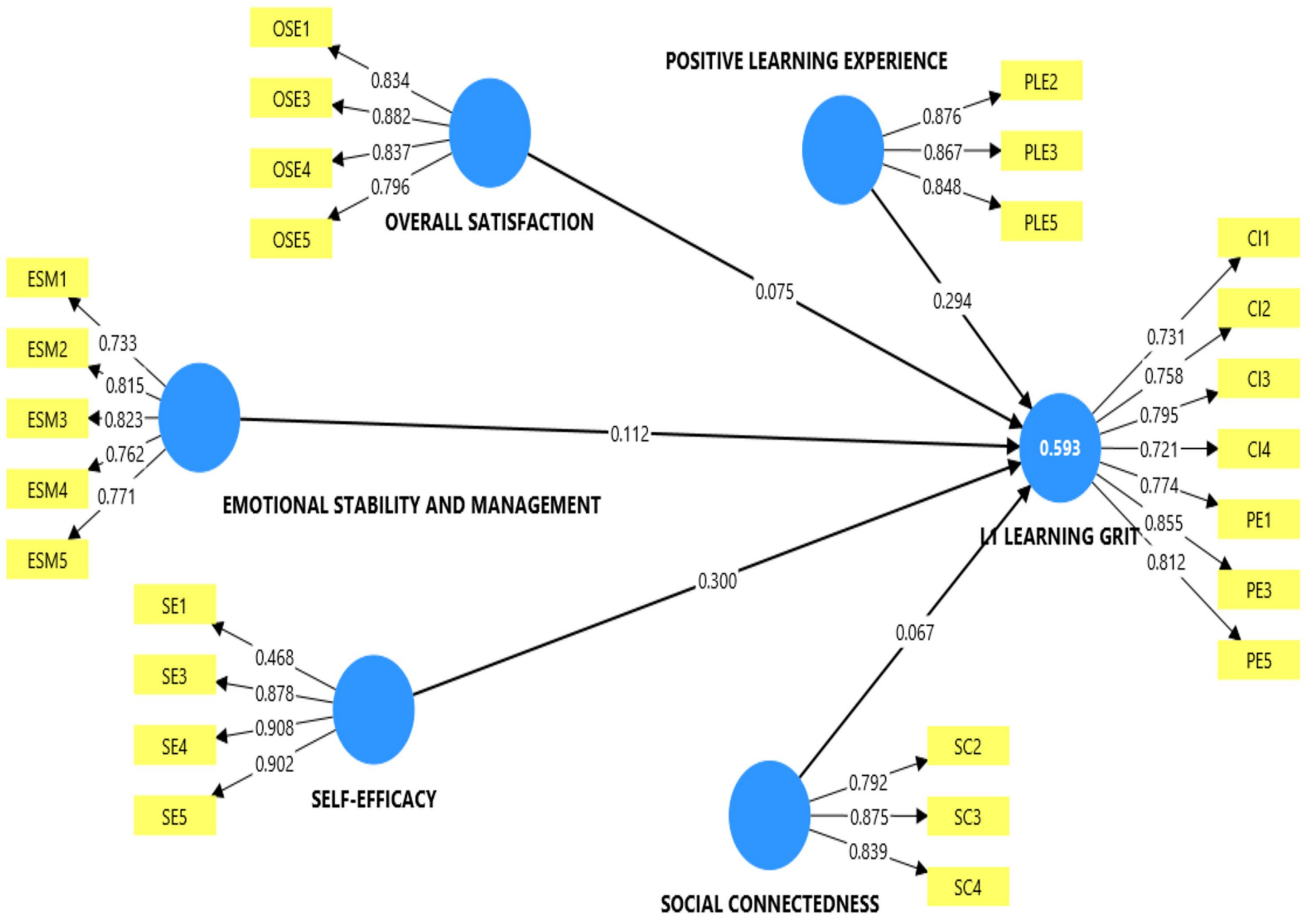
PLS-SEM algorithm results.

With regard to the reliability analysis, the first consideration is the quality of the indicator loadings. Indicator loading of 0.7 or higher demonstrates a higher level of internal consistency in the measure of the constructs. Based on this criterion most of the indicator loadings were removed because they loaded recommended threshold and also affected appropriateness of the average variance extracted (AVE) values. Two indicators (PLE 1 and PL6) which loaded below 0.7 were removed from the positive learning experience construct. Also, SE5 and SE6 on self-efficacy and SC1, SC5 and SC6 on the social connectedness construct were also removed. These items were removed because their inclusion in their respective constructs compromised the stability of their AVE values. The remaining indicators that loaded below this threshold in other constructs such as the LLG and self-efficacy construct were retained because, as scholars have argued, loading below this threshold (as low as 0.4) could be retained in a model when they do not affect other thresholds in the model, especially the AVE values. Also, Cronbach’s alpha and the composite reliability (Rho_A and Rho_c) of the current model support the existence of internal consistency in the measurements as they meet the 0.7 or higher threshold recommended by scholars (Hair et al., 2019). These results are presented in Table 2 and Figure 1.

**Table 2:** Model reliability and convergent validity.

Constructs	Loadings	*α*	rho_A	CR	AVE
LLG		0.891	0.895	0.915	0.607
CI1	0.731				
CI2	0.758				
CI3	0.795				
CI4	0.721				
PE1	0.774				
PE3	0.855				
PE5	0.812				
ESM		0.841	0.847	0.887	0.611
ESM1	0.733				
ESM2	0.815				
ESM3	0.823				
ESM4	0.762				
ESM5	0.771				
OSE		0.859	0.868	0.904	0.702
OSE1	0.834				
OSE2	0.882				
OSE3	0.837				
OSE4	0.796				
OSE5	0.834				
PLE		0.829	0.829	0.898	0.746
PLE2	0.876				
PLE3	0.867				
PLE5	0.848				
SCON		0.785	0.791	0.874	0.699
SC2	0.792				
SC3	0.875				
SC4	0.839				
SEF		0.808	0.871	0.879	0.657
SE1	0.468				
SE3	0.878				
SE4	0.908				
SE5	0.902				

The convergent validity —which measures the extent to which a set of indicators are reflective of a common underlying construct (Hair et al., 2019)—was assessed using the AVE. As a rule of thumb, AVE values of 0.5 or higher are indicative of the fact that a set of items converge adequately to measure a common construct. This requirement has also been met in this study because the AVE of each of the constructs in the current mode is above the recommended threshold, confirming that the items of the constructs are indeed converging on a common construct.

#### 4.2.2 Discriminant validity

Discriminant validity was also evaluated to ensure that each of the constructs in the model are unique in themselves and share less variance with the indicators of other constructs. In simple terms, the discriminant validity analysis was used to ensure that the constructs in the model differ significantly from each other. To statistically ascertain this, the Heterotrait–Monotrait ratio (HTMT) and the Fornell–Larcker criterion were employed. The results of the discriminant validity based on the threshold are presented in Table 3.

**Table 3:** Discriminant validity.

HTMT ratio	ESM	LLG	OSE	PLE	SE	SC
ESM						
LLG	0.686					
OSE	0.741	0.740				
PLE	0.592	0.447	0.408			
SE	0.754	0.443	0.676	0.407		
SC	0.575	0.776	0.590	0.666	0.918	

Fornell–Larcker criterion						
	ESM	LLG	OSE	PLE	SE	SC
ESM	0.782					
LLG	0.600	0.779				
OSE	0.615	0.654	0.838			
PLE	0.569	0.633	0.765	0.864		
SE	0.635	0.526	0.750	0.648	0.810	
SC	0.615	0.654	0.731	0.581	0.443	0.836

The HTMT ratio establishes the conceptual distinction between constructs when the correlation between indicators of the same construct is stronger than the correlation between indicators across constructs (Henseler et al., 2015). HTMT values below 0.9 is an indication that there is discriminant validity in the model. Thus, it could be concluded that the current model has discriminant validity as none of the HTMT values surpass 0.9. The Fornell–Larcker criterion also provides further evidence to support the discriminant validity of the current study. With the Fornell-Larcker criterion, discriminant validity is established when the square root of the AVE of each of the constructs are higher than the squared correlations among the constructs (Hair et al., 2019; Henseler et al., 2015). The Fornell-Larcker criterion results for this study aligns with this recommendation, hence supporting the fact that the constructs in this model are conceptually distinct.

#### 4.2.3 Common method bias

Another important quality check in the measurement model is the common method bias (CMB). CMB becomes an issue in PLS-SEM when a common measurement is used to gather data on both criterion and predictor variables, which could possibly lead to inflated correlation among the predictor variables and the criterion, and consequently culminate in biased hypothesis results (Kock, 2015). It is therefore recommended to check the prevalence of CMB in a model through the multicollinearity assessment or Harman’s single factor test. In this study, we employed the multicollinearity procedure to ascertain whether there are issues of CMB. The multicollinearity was assessed through the variance inflation factor (VIF). As Kock (2015) suggest a model is devoid of multicollinearity issues when the values of the VIF for the predictor variables are below 3.3. Based on this requirement, the current model have no issue of CMB since none of the VIF values (see Table 4) surpass 3.3, hence the results of the hypotheses are deemed reliable.

**Table 4:** Common method bias.

Constructs	ESM	LLG	OSE	PLE	SE	SCON
ESM		2.465				
LLG						
OSE		2.211				
PLE		2.635				
SE		2.011				
SC		3.003				

### 4.3 Structural model assessment

**Figure 2:**
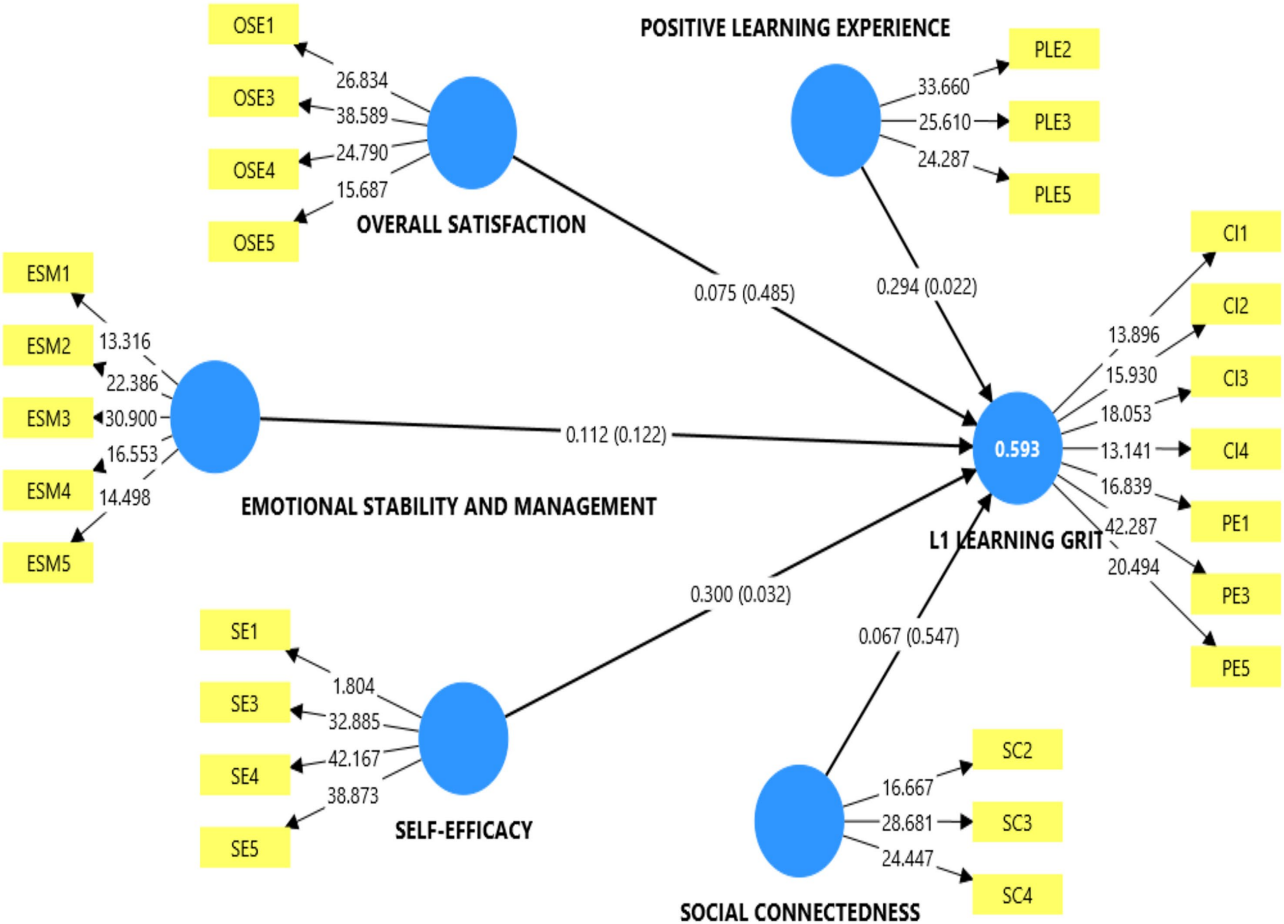
PLS-SEM bootstrapping results.

The structural model assessment was focused on testing the hypothesis that students’ psychological wellbeing will have a significant positive influence on the LLG among university students pursuing Ghanaian languages. Specifically, we tested the influence of the positive learning experience (PLE), overall satisfaction and enjoyment (OSE), emotional stability and management (ESM), social connectedness (SCON), and self-efficacy (SEF) on their LLG. The major statistical values that were used to test these hypotheses included the path coefficient (*β*), a measure of the direction and strength of association between, the *t* and *p*-values (the significance levels), the effect size (*f*^2^) and the coefficient of determination (*R*^2^). Table 5 and Figure 2 presents the results of the hypotheses.

**Table 5:** Structural model hypothesis result.

Hypotheses	Paths	*Β*	SD	*T*	*P*	*F* ^2^	*R* ^2^	*Q* ^2^	Decision
H1	ESM → LLG	0.112	0.072	1.548	0.122	0.033	0.593	0.431	Reject
H2	OSE → LLG	0.075	0.108	0.698	0.485	0.000			Reject
H3	PLE → LLG	0.294	0.128	2.291	0.022	0.051			Accept
H4	SCON → LLG	0.067	0.111	0.603	0.547	0.006			Reject
H5	SEF → LLG	0.300	0.140	2.140	0.032	0.075			Accept

The results of the hypotheses confirmed that while some subconstructs of psychological wellbeing are significant predictors of the learning grit of Ghanaian language students, others did not have any compelling predictive influence on their LLG. The results of the PLS-SEM bootstrap with 5,000 subsamples indicated the LLG of students was positively influenced by students’ PLE (*β* = 0.294; *t* = 2.291; *p* = 0.022; *f*^2^ = 0.051), and SEF (*β* = 0.300; *t* = 2.140; *p* = 0.032; *f*^2^ = 0.075), hence confirming H3 and H5, respectively. The associated effect sizes for the confirmed hypothesis indicate that the statistically significant positive influence of these psychological well-being variables on the LLG of students is quite minimal. As Cohen (1998) suggests, effect sizes of *f*^2^ = 0.02, 0.15, and 0.35 are indicative of small, medium, and large statistical impact, respectively. The practical implication of these confirmed hypotheses is that the students’ consistency of interest in learning Ghanaian languages, their perseverance of effect are likely to heighten or improve whenever there is a substantial improvement in the level of satisfaction and enjoyment of learning, the emotional stability and management as well as sense of efficacy in learning.

Despite the insights above, the rest of the hypotheses were not found to be predictors of students’ L1 learning grit. This means that a student’s language learning efforts or the magnitude of efforts they put in learning Ghanaian languages does not depend on their social connectedness, emotional stability and management, and their overall enjoyment and satisfaction with learning. This presupposed that an increase in these variables is not likely to increase their L1 learning grit.

The coefficient of determination was also assessed to determine the magnitude of variance in students LLG accounted for by the predictor variables. As can be seen in Table 5 the *R*^2^ value of 0.652 indicates that the predictors accounted for 59.3% of the variance in L1 learning grit. According to Hair et al. (2019), *R*^2^ values of 0.25, 0.50, and 0.75 can be interpreted as weak, moderate and substantial, respectively. Hence, the current models’ *R*^2^ of 0.593 shows that the predictors accounted for almost a substantial proportion of variance on the endogenous variable. Following the *R*^2^ assessment, the predictive relevance was run using the blindfold cross-validated redundancy procedure to test the accuracy of the prediction. According to Hair et al. (2019), a model demonstrates predictive accuracy when the *Q*^2^ value is 0 or more. Since the *Q*^2^ of the current model surpassed 0, we conclude that the current model offered an accurate prediction which confirms the robustness of the results.

## 5 Discussion

The influence of psychological well-being on the academic grit of students has received considerable attention in the literature (Al-Zain and Abdulsalam, 2022; Harpaz et al., 2024). In relation to language learning, much of the scholarly discourses have focused solely of L2 grit among learners. In relation to L1, especially in the context of African language learning at the university level, there seems to be a knowledge gap which makes the current study a significant one. The finding of the current study filled this scholarly void by looking closely at how university students’ psychological well-being predicts their LLG in the Ghanaian context.

The current study reveals a strong connection between positive learning experiences and LLG among students in the university. It has shown the more students are motivated by the success they achieve in their learning of the Ghanaian languages, the more they invest their efforts and interest in learning. This means that the efforts students of the various Ghanaian languages invest in their studies is partly dependent on the positive experiences they get in their learning, which presupposes that if students are faced with negative learning experiences, the grit in learning L1 at the university level could diminish over time. The current findings support the crucial of positive psychology on students’ academic engagement (Morales-Rodríguez et al., 2020; Zhang et al., 2020). It reinforces the need to create a positive language learning environment because the results have shown that language students rely on the experience they gain in learning to reinforce their academic engagements and efforts.

Finally, the study has shown the self-efficacy related to learning the L1 at the university level has a compelling predictive influence on their grit. This result means that the more students believe in their ability to excel in learning the native languages at the university, the stronger the magnitude of effort and interest they put in L1 learning. In plain language, the results indicate that students who think they can excel in language learning set language learning goals, and try all means possible overcoming all barriers to achieving their language learning goals. It could therefore be argued that students who are less efficacious about the ability to excel in L1 learning at the university level would be less gritty and are likely to relent in setting L1 learning targets, as well as working toward achieving these targets. Zheng et al. (2022) reported a similar finding in their study of emotion regulation, self-efficacy, and L2 grit in higher education. In consonance with the current study, they reported that self-efficacy is a stronger predictor of L2 grit, which means the stronger students’ confidence regarding their ability in learning English as a foreign language, there high their academic grit. The study of Yang et al. (2024) also confirms the predictive impact of self-efficacy in the language learning grit of students in Iran and China.

The study has however indicated that psychological well-being constructs including overall satisfaction and enjoyment, emotional stability and social connectedness does not influence the language learning grit of students. These findings contradict the existing literature like that of Tangmunkongvorakul et al. (2022) and Grisier (2018). The lack of consonance between the current study’s findings and existing studies probably stems from the contextual discrepancy. The lack of association between satisfaction and enjoyment and social connectedness could be tied to how students perceive the usefulness and significance of learning Ghanaian languages at the tertiary level. In the Ghanaian context pursuing Ghanaian languages at the university level is less regarded by peers (Edu-Buandoh and Otchere, 2012; Nyamekye, 2024). Hence, gritty language students who express deeper enthusiasm in pursuing L1 at the university level are likely to be labeled. This has a detrimental impact on the sense of belonging, social connectedness, satisfaction, and enjoyment. This possibly explains why social connectedness and overall enjoyment emerge as the only psychological well-being variables that did not influence the grit of students.

## 6 Implications of the study

Having revealed that students’ psychological well-being has a compelling influence on the LLG, there is a need to offer constructive suggestions that can be used to shape policy and practical decisions as far as L1 education is concerned in Ghana. Foremost, there is a need for targeted interventions that foster the emotional stability of students in pursuing Ghanaian languages in Ghana. Achieving this requires dealing with the root causes of the negative emotional stability associated with learning the indigenous languages of Ghana. This involves the prohibition of unfavorable educational practices at the basic levels of education in Ghana, especially the punishment and embarrassment of students for speaking their native languages in school. Such practices at the early levels of education shape children’s conception of the value of learning their L1, which manifests itself later at the higher levels of education in Ghana. Effective bilingual education models (especially dual language bilingual education) which place equal importance on languages could be emphasized throughout the lower level of education to reshape students’ perceptions of the value of their L1. This could help enhance the emotional responses of students as L1 learning as they get to the higher levels of education.

There is a need for effective institutional support systems that could enhance students’ L1 learning self-efficacy and their resilience development. This could be in the form of mentorship and counseling programs. Anecdotal records show that most students pursuing L1 as a program of study lack enough knowledge of the career opportunities in the field. Through guidance and counseling, students would get to appreciate the essence of L1 education, which can consequently build the confidence and effort they put into their studies to achieve their learning goals. Universities must also work toward reducing the stigma associated with studying Ghanaian languages by promoting awareness campaigns and encouraging faculty and peer support. Lastly, increased institutional investment in resources, scholarships, and career pathways for Ghanaian language students will further motivate them and reinforce the value of L1 education in national development.

## 7 Limitation and further studies

Despite the efforts in the current study, there are limitations to the current study which can serve as the basis for further explorations into the issue. Foremost the study employed a purely quantitative approach and as a limitation to quantitative research, the subjective experience of students with regard to their psychological well in language learning was adequately not captured. We, therefore, recommend an interpretive phenomenological study to address this gap. Another limitation is that the study focused solely on higher education learners. We therefore recommend that further studies should look into the well-being and LLG among high school learners in Ghana.

## Data Availability

The raw data supporting the conclusions of this article will be made available by the authors, without undue reservation.
